# Short- and Long-Term Cognitive Effects of Chronic Cannabinoids Administration in Late-Adolescence Rats

**DOI:** 10.1371/journal.pone.0031731

**Published:** 2012-02-13

**Authors:** Hila Abush, Irit Akirav

**Affiliations:** Department of Psychology, University of Haifa, Haifa, Israel; Hokkaido University, Japan

## Abstract

The use of cannabis can impair cognitive function, especially short-term memory. A controversial question is whether long-term cannabis use during the late-adolescence period can cause irreversible deficits in higher brain function that persist after drug use stops. In order to examine the short- and long-term effects of chronic exposure to cannabinoids, rats were administered chronic i.p. treatment with the CB1/CB2 receptor agonist WIN55,212-2 (WIN; 1.2 mg/kg) for two weeks during the late adolescence period (post-natal days 45–60) and tested for behavioral and electrophysiological measures of cognitive performance 24 hrs, 10 and 30 days after the last drug injection. The impairing effects of chronic WIN on short-term memory in the water maze and the object recognition tasks as well as long-term potentiation (LTP) in the ventral subiculum (vSub)-nucleus accumbens (NAc) pathway were temporary as they lasted only 24 h or 10 d after withdrawal. However, chronic WIN significantly impaired hippocampal dependent short-term memory measured in the object location task 24 hrs, 10, 30, and 75 days after the last drug injection. Our findings suggest that some forms of hippocampal-dependent short-term memory are sensitive to chronic cannabinoid administration but other cognitive impairments are temporary and probably result from a residue of cannabinoids in the brain or acute withdrawal effects from cannabinoids. Understanding the effects of cannabinoids on cognitive function may provide us with tools to overcome these impairments and for cannabinoids to be more favorably considered for clinical use.

## Introduction

Cannabis is the most widely used illicit drug after nicotine and alcohol [1] and can impair several aspects of cognitive function [2], [3].

In humans, cannabinoids impair both encoding and recall of verbal and non verbal information depending on dose and task difficulty [2]. In animal studies, cannabinoids impair memory in a variety of experimental conditions such as the radial maze, instrumental discrimination tasks and the Morris water maze [4].

Given the well established role of the hippocampus in learning and memory processes, and its high expression of CB1 receptors [5], [6], it is likely that the adverse effects of cannabinoids on spatial learning tasks, short-term memory, and attention are attributable to their actions within this brain region. Using electrophysiological recordings from hippocampal slices, previous studies have shown that cannabinoid receptor activation inhibits LTP in the hippocampus [7]–[11]. We have shown that acute administration of the CB1/2 receptor agonist WIN55,212-2 (WIN; 0.5 mg/kg) impairs the induction of LTP in the schaffer collateral-CA1 projection of anesthetized rats [12]. Additionally, WIN administered systemically or into the CA1 (5 µg/side) impairs spatial learning in the water maze [12]. Hence, acute exposure to cannabinoids impairs both hippocampal spatial learning and LTP. Recently, we found that acute WIN administered into the ventral subiculum (vSub; 5 µg/side) also impairs acquisition and retrieval of memory in the social discrimination task [13].

A controversial question is whether long-term exposure to cannabinoids can cause irreversible deficits in higher brain function that persist after drug use stops. Cognitive deficits caused by long term exposure to cannabinoids can last for many days, and possibly for weeks [14]–[16], after discontinuing use, but it is still unclear whether long-term cannabinoids exposure causes irreversible cognitive deficits.

Results from studies in humans [1] and rats [17], [18] suggest that vulnerable periods exist during human and rat brain development up to the age of 16 years (post-natal day (PND) ∼35–40 in rats), during which cannabis can permanently compromise cognitive functions.

It is difficult to define the time course of adolescence, with no single event signaling its onset or termination [19]. During adolescence, the brain undergoes numerous changes [20]; massive loss of synapses in neocortical regions, remodeling of the prefrontal cortex, maturational changes in the hippocampus [20]–[22]. Neuroplastic modifications also include changes in dendritic spine density, synaptic rearrangements and development of myelination [23]. This remodeling process may be disrupted by cannabinoids leading to lasting adverse effects on brain and behavior [23]. Receptors for endogenous cannabinoids mature slowly during the postnatal period [24], [25], with binding peaking during adolescence at higher than adult levels in hippocampus [25]. These endogenous cannabinoid systems may reach functional maturity around adolescence [26], [27].

Evidence from both animal and human studies suggests that frequent exposure to cannabis during adolescence may have long-term effects on the development of cognition, brain structure and function [14], [15], [28], [29], [30]. However, studies on adults were not straightforward in determining whether such deficits, observed after only hours or days of abstinence, are temporary (perhaps due to a residue of cannabinoids in the brain) or long-lasting (due to a neurotoxic effect of long-term exposure). For example, Quinn et al., [23] found deficits in object recognition following repeated exposure to Δ^9^-THC (THC) in adolescent but not adult rats, a result consistent with other reports of cannabinoid administration in immature but not mature rats causing lasting impairments in learning [17], [18], [28].

In the current study, we aimed to examine the short- and long-term effects of chronic exposure to cannabinoids in the late-adolescence period that falls between adolescence and adulthood (PND ∼45–60). To that end, male rats were chronically injected with a cannabinoid receptor agonist during late-adolescence and behavioral and electrophysiological measures of cognitive performance were tested 24 hours, 10 and 30 days after cessation of drug treatment.

## Materials and Methods

### Subjects

Male Sprague-Dawley rats (45 days old, ∼200 g; Harlan, Jerusalem, Israel) were caged together (5 per cage) at 22±2°C under 12-hour light/dark cycles (lights turned on at 07:00 and turned off at 19:00). Rats had access to water and laboratory rodent chow *ad libitum*. The experiments were approved by the University of Haifa Ethics and Animal Care Committee, and adequate measures were taken to minimize pain or discomfort.

### Drug Treatment

The CB1/2-receptor agonist WIN55,212-2 (Tocris, USA) was initially dissolved in dimethylsulfoxide (DMSO), and further diluted with 1% Tween 80 and 98% saline (0.9% NaCl). Final DMSO concentration was 1%. This DMSO and saline solution was also used as the vehicle.

WIN was administered intraperitoneally (i.p.) at a dose of 1.2 mg/kg, 0.3 ml (based on previous reports [31], [32]). For chronic experiments, WIN was administered during PND 45–60, as the rats received 14 injections i.p., one per day. For acute experiments, WIN was administered in a single i.p. injection (1.2 mg/kg) 24 h before testing.

### The Morris Water Maze Task

The water maze, placed in a dimly lit room, consisted of a pool of water (diameter 1.7 m; 50 cm high rim; manufactured by the University of Haifa). For the spatial training task a submerged escape platform (12×12 cm) was placed 30 cm away from the edge in a fixed location. Each trial was initiated by placing the animal in one of three quadrants (in which there is no platform) near the wall of the tank. Animals were allowed to search for the hidden platform for a maximum of 60 s, while their latency to find the hidden platform was manually recorded by an experimenter. If a rat did not reach the platform within 60 s, an experimenter would guide it there [12]. The rat was then allowed to remain on the platform for 25 s before removal back to the home cage.

**Figure 1:**
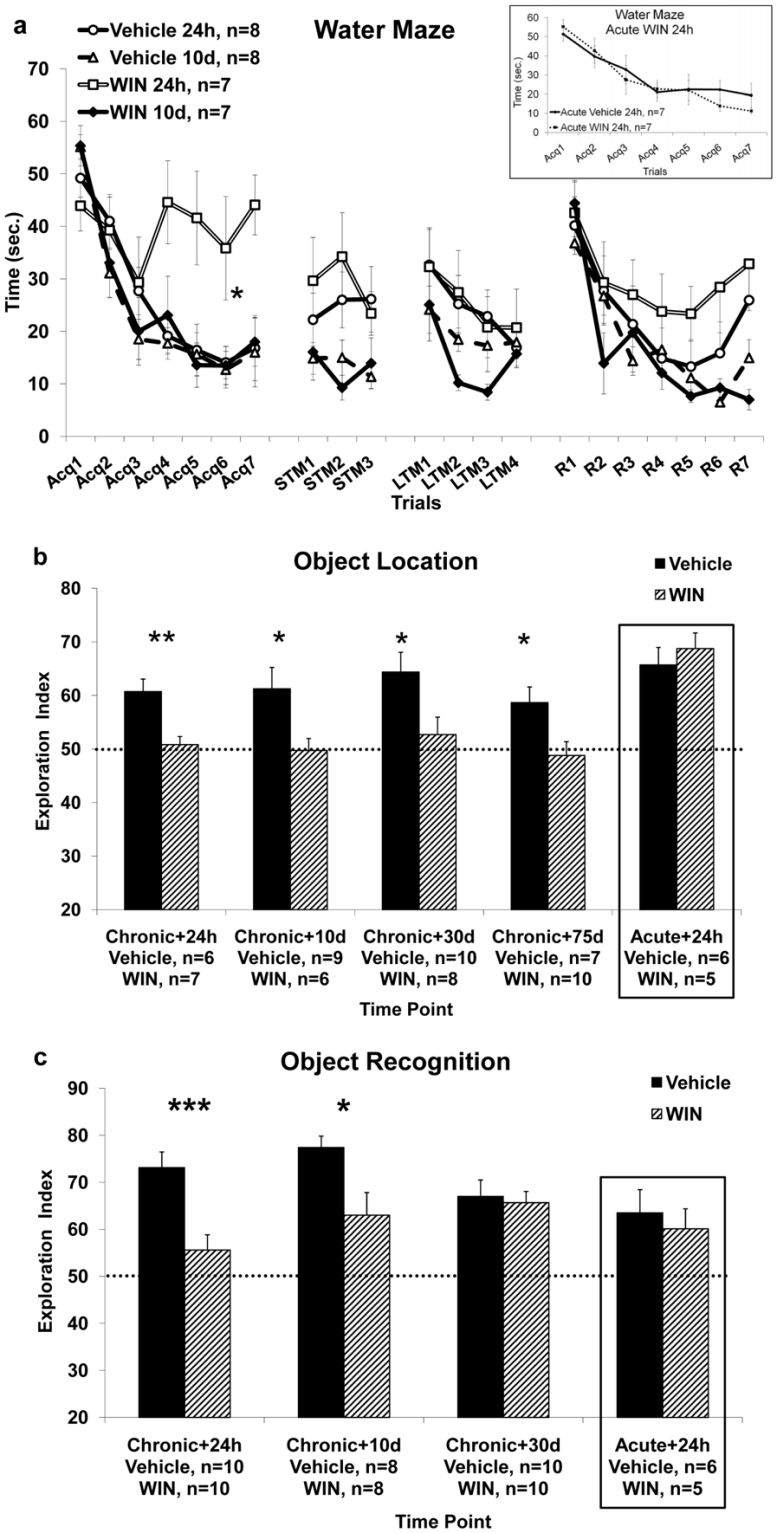
The effects of chronic exposure to WIN55,212-2 during late-adolescence on hippocampal and non-hippocampal dependent tasks. **1a.** Rats tested in the water maze 24 h, but not 10 d, after the last WIN injection, show increased latency to locate the hidden platform compared with the vehicle group on the first training day (Acq1–7). (*, p<0.05: WIN 24 h different from Vehicle 24 h). **Inset:** a control experiment where rats were tested in the water maze 24 hours following a single WIN injection. **1b.** Rats tested in the object location task 24 h, 10 d, 30 d and 75 d after the last vehicle injection spent significantly more time exploring the new location compared with the WIN groups. (*, p<0.05; **, p<0.01: Vehicle different from WIN). **On the right square**: a control experiment where rats were tested in the object location task 24 hours following a single WIN injection. **1c.** Rats tested in the object recognition task 24 h or 10 d, but not 30 d, after the last vehicle injection spent significantly more time exploring the new location compared with the WIN groups (*, p<0.05; ***, p<0.001: Vehicle different from WIN). **On the right square**: a control experiment where rats were tested in the object recognition task 24 hours following a single WIN injection.

The experiment consisted of 3 days (**Figure 1a**). All trials are presented in blocks of two. On the *first day*, the animals went through a massed training protocol of 14 trials with inter-trial intervals of 3 min [**Acq_1–7_**] [33], and 6 more trials to assess short-term memory conducted after 30 min [**STM_1–3_**]. On the *second* day, the animals underwent 8 trials to further train them before assessing their performance in a reversal task, as the massed protocol is considered to be less efficient in producing learning than the spaced paradigms often used in other studies [33] [**LTM_1–4_**]. This training session can also be a measure of long-term memory retrieval as rats that have acquired the task will demonstrate better performance. On the *third* day, the platform was moved to the opposite quadrant of the maze. The animals went through 10 reversal trials in which they were tested for their ability to learn the new platform location [**R_1_–R_5_**].

### Object location memory task

This task measures an animal's ability to detect that an object has moved to a new location. This is a hippocampal-dependent spatial memory task [34]–[36]. The objects were two small identical ceramic dolls (10×8×7 cm; painted blue and pink) located in a squared black open-field (50×50×50 cm) under dim light, 10 cm from the walls. The open-field and the objects were thoroughly cleaned between trials with odorous clean wipes.

The rats were habituated to the experimental apparatus by allowing them to explore it for 10 min every day for 4 days without objects before the experiment was performed. In the sample phase, each rat was placed in the open-field arena and exposed to the objects for 5 min. The test phase was given 30 min after the sample trial (i.e., to test short-term memory). One object was moved to a new location and the time spent exploring the objects at the old and new locations were recorded for 5 min.

A digital camera placed above the arena and connected to a video tape was used to track rat behavior during the exploration session. Recorded data was analyzed by two judges blind to experimental conditions and inter-rater reliability was assured.

**Figure 2:**
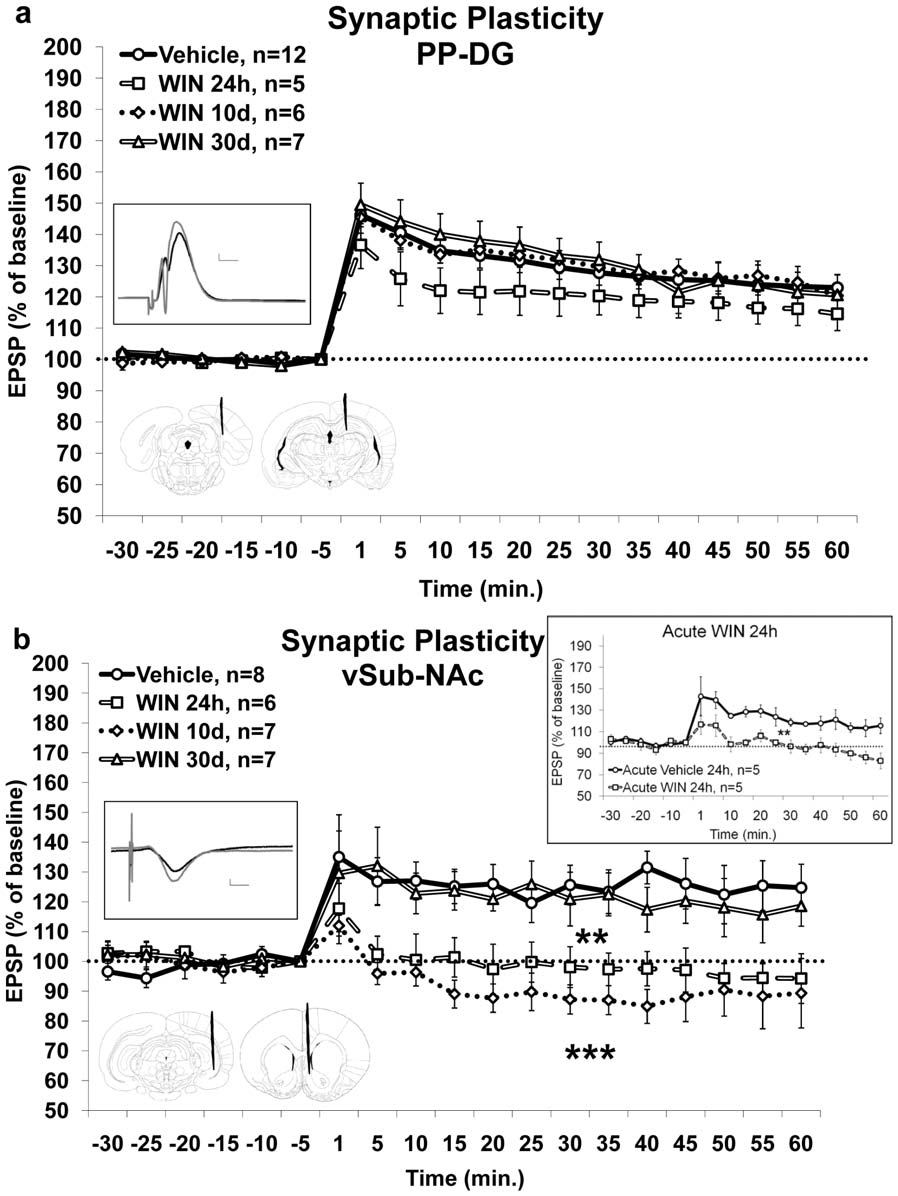
The effects of chronic exposure to WIN55,212-2 during late-adolescence on synaptic-plasticity in the hippocampus and nucleus-accumbens. **2a.** Chronic administration of WIN had no effect on LTP levels in the PP-DG pathway measured 24 h, 10 d or 30 d after the last drug injection. **Inset:** **Up:** representative traces in the DG for the vehicle group taken before (black) and 5 min after (gray) HFS to the PP (calibration: 0.2 mV, 10 µs); **Down:** schematic drawing of electrodes tip positions: left - representative location of the stimulating electrode tip in the perforant path (anteroposterior, −8.0 mm; lateral, ±4.0 mm; ventral, −3.0 mm), right - representative location of the recording electrode tip in the dentate gyrus (anteroposterior, −4.0 mm; lateral, ±2.5 mm; ventral, −3.7 mm). **2b.** Chronic administration of WIN impaired LTP levels in the vSub-NAc pathway measured 24 h or 10 d, but not 30 d, after the last drug injection. (**, p<0.01: WIN 24 h group different from WIN 30 d and Vehicle groups, and WIN 10 d group different from WIN 30 d group; ***, p<0.001: WIN 10 d group different from Vehicle group). **Inset:** **Up:** representative traces in the NAc for the vehicle group taken before (black) and 5 min after (gray) HFS to the vSub (calibration: 0.2 mV, 10 µs); **Down:** schematic drawing of electrodes tip positions: left - representative location of the stimulating electrode tip in the ventral subiculum (anteroposterior, −6.5 mm; lateral, ±5.0 mm; ventral, −6.0 mm), right - representative location of the recording electrode tip in the nucleus accumbens (anteroposterior, +1.6 mm; lateral, ±1.0 mm; ventral, −5.5 mm); **On the right:** a control experiment where acute administration of WIN impaired LTP levels in the vSub-NAc pathway measured 24 h after a single injection (**, p<0.01).

Exploration was defined as when the subject sniffed at, whisked at, or looked at the object from no more than 2 cm away. An exploration index calculated for each animal was expressed as *T*_N_/(*T*_N_+*T*_F_) (*T*_F_ = time spent exploring the object in the familiar location; *T*_N_ = time spent exploring the object in the novel location). Intact spatial recognition memory in the test phase was reflected in an exploration score higher than 0.5, which implies greater exploration of the object in the novel location (**Figure 2a**).

### Object recognition memory task

This task measures the ability to discriminate the familiarity of previously encountered objects. If a rat is presented with both a familiar object and a novel object, it will direct more exploration at the novel object. This task is dependent on the prefrontal cortex and perirhinal cortex [37], [38]. In the sample phase, each rat was placed in the open-field arena and exposed to two identical objects (the same objects as in the object location memory task) for 5 min. In the test phase, thirty min after the sample trial, the rat was presented with one of the objects from the sample trial and with a novel object (ceramic triangle, 10.5×5×2 cm; painted gray) for 5 min (**Figure 1c**). The familiar and novel objects were counterbalanced during the sample and test phases. The rest of the parameters were identical to the object location task described above.

### Electrophysiology

#### Surgical Procedure

Rats were anesthetized (with 40% urethane, 5% chloral hydrate in saline, injection volume of 4 ml/1 kg, i.p.) and placed in a stereotaxic frame. Small burr holes were drilled in the skull to allow electrodes to be inserted into the brain. A recording microelectrode (glass, tip diameter of 2–5 µm, filled with 2 M NaCl, resistance of 1–4 M) was inserted into the DG (anteroposterior, −4.0 mm; lateral, ±2.5 mm; ventral, −3.7 mm) (**Figure 2a**) or into the NAc shell (anteroposterior, +1.6 mm; lateral, ±1.0 mm; ventral, −5.5 mm) (**Figure 2b**). A bipolar 125 µm stimulating electrode was positioned in the perforant path (PP; anteroposterior, −8.0 mm; lateral, ±4.0 mm; ventral, −3.0 mm) (**Figure 2a**) or the vSub (anteroposterior, −6.5 mm; lateral, ±5.0 mm; ventral, −6.0 mm), respectively (**Figure 2b**). After positioning the electrodes, the rat was left for 60 minutes before commencing the experiment.

#### LTP Induction

LTP was induced by theta-like high-frequency stimulation (HFS) (three sets of 10 trains; each train consisting of 10 pulses at 200 Hz; inter-train interval, 200 ms; inter-set interval, 1 min) to the vSub or PP. Field potentials were recorded from the NAc or DG every 5 minutes for 60 minutes after HFS to the vSub/PP. LTP was measured as an increase in the amplitude of the excitatory post-synaptic potentials (EPSPs). Potentiation was measured as a percentage change from the average of the 30 min baseline before HFS.

### Open Field

The apparatus consisted of a square black open-field (50×50×50 cm). The floor was divided by 1-cm-wide white lines into 25 squares measuring 10×10 cm each. A video image of the open-field was displayed on a TV monitor, and the movements of the rat were manually recorded by two ‘blind’ experimenters and analyzed in order to measure motor activity over a period of 5 min.

**Figure 3:**
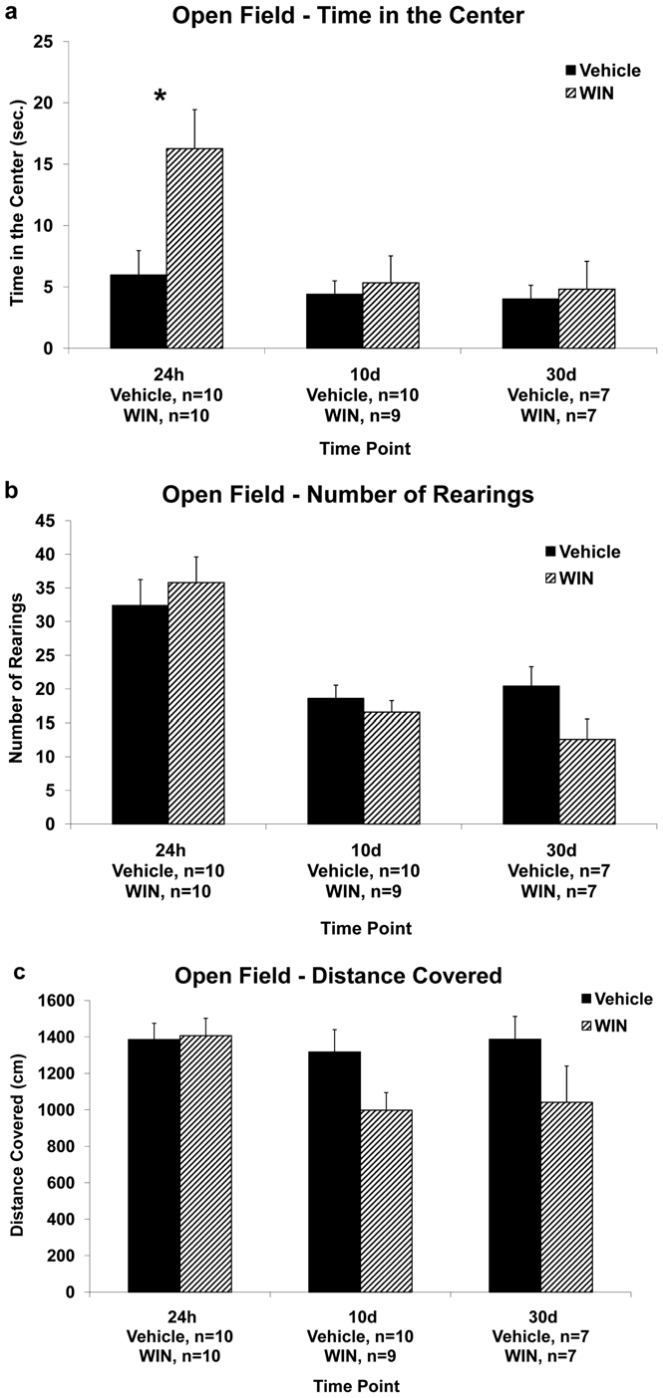
The effects of chronic exposure to WIN55,212-2 during late-adolescence on locomotion and anxiety in the open-field. **3a.** Chronic administration of WIN caused an increase in the time the rats spent in the center when tested in the open field 24 h after the last drug injection. (*, p<0.05: Vehicle different from WIN). **3b.** Chronic administration of WIN had no effect on the number of rearings the rats performed in the open field when measured 24 h, 10 d or 30 d after the last drug injection. **3c.** Chronic administration of WIN had no effect on the distance the rats covered in the open field when measured 24 h, 10 d or 30 d after the last drug injection.

Recordings were made of the time the rat spent in the central and peripheral squares, the number of instances of rearing, and the total distance covered (**Figure 3**). The open-field arena was thoroughly cleaned between trials with odorous clean wipes.

### Sucrose intake

**Figure 4:**
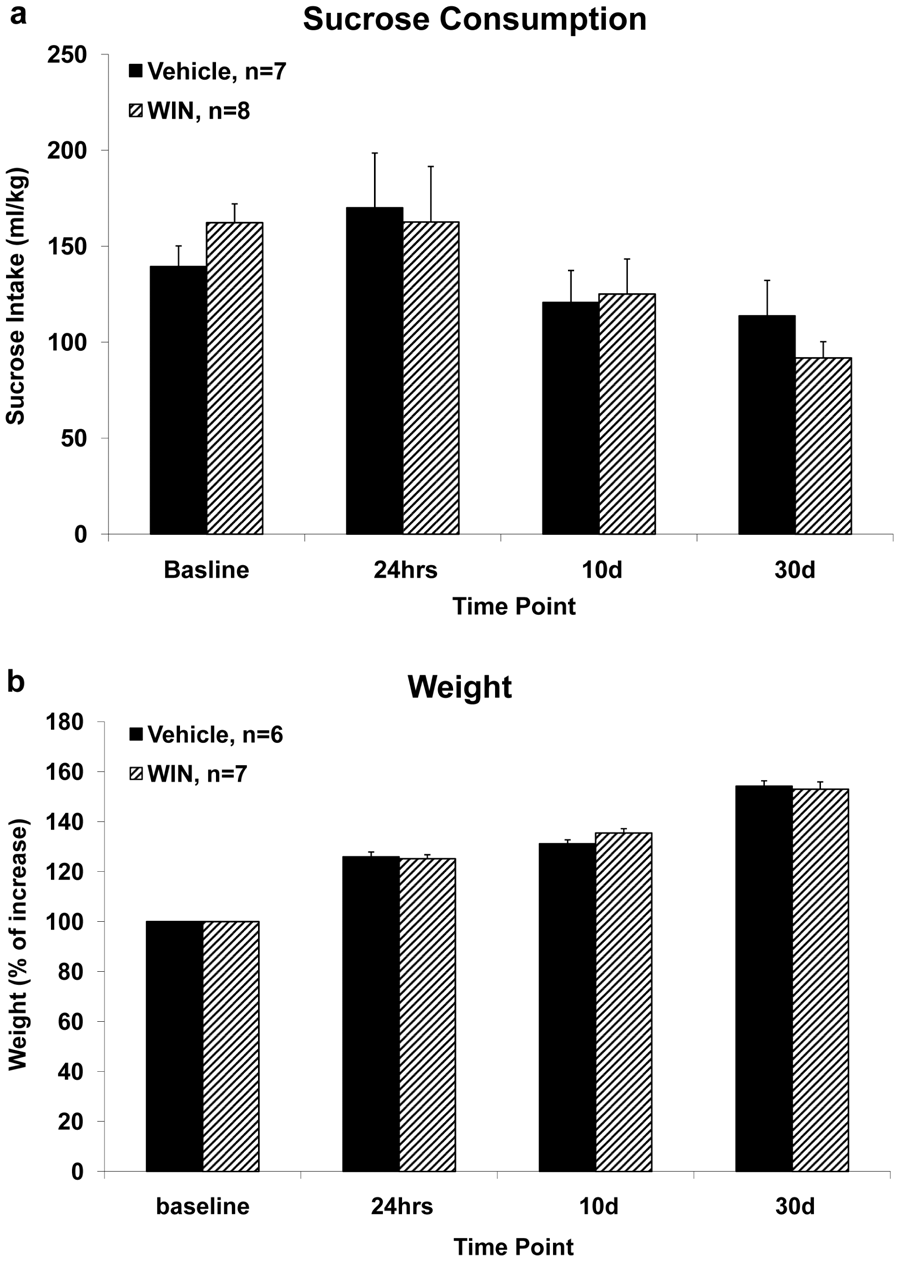
The effects of chronic exposure to WIN55,212-2 during late adolescence on sucrose consumption. **4a.** Chronic administration of WIN had no effect on sucrose consumption when measured at baseline, 24 h, 10 d or 30 d after the last drug injection. **4b.** Chronic administration of WIN had no effect on weight gain compared to the vehicle group when measured at baseline, 24 h, 10 d, or 30 d after the last drug injection.

Water bottles were removed before the dark part of the cycle, and replaced with bottles containing a 1% sucrose solution. Sucrose consumption was measured during the 12 dark hours of the cycle and was then normalized according to every rat's specific weight (**Figure 4a**). Rats were individually housed during the sucrose intake measurement.

### Statistical Analysis

The results are expressed as means ±SEM. For statistical analysis, mixed design ANOVA, 3-way mixed ANOVA, 2-way ANOVA, and t-test were used as indicated. All *post hoc* comparisons were made using the least significant difference multiple-comparison test (LSD).

### Experimental design

Rats were chronically injected for 14 days with WIN or vehicle. Every rat underwent one behavioral or electrophysiological test, to prevent carryover effects due to multiple tests. However, the sucrose consumption and weight gain measures were taken from rats that were tested behaviorally or electrophysiologically. Hence, different groups of rats were tested at the different time points post-injection.

**Behavioral measures:** (i) water maze– 24 h or 10 days after the last chronic injection and 24 h after a single injection (figure 1a), (ii) object location task– 24 h, 10, 30 or 75 days after the last chronic injection and 24 h after a single injection (figure 1b), (iii) object recognition task– 24 h, 10 days or 30 days after the last chronic injection and 24 h after a single injection (figure 1c).

**Electrophysiological measures:** (i) LTP in the PP-DG pathway– 24 h, 10 days or 30 days after the last injection (figure 2a), (ii) LTP in the vSub-NAc pathway– 24 h, 10 days or 30 days after the last chronic injection and 24 h after a single injection (figure 2b).

**Control experiments:** (i) locomotion and anxiety-like behavior in the open field– 24 h, 10 days or 30 days after the last injection (figure 3), (ii) sucrose consumption– 24 h, 10 days or 30 days after the last injection (figure 4a), and (iii) weights– 24 h, 10 days or 30 days after the last injection (figure 4b).

## Results

### The effects of chronic exposure to the cannabinoid receptor agonist WIN55,212-2 during late adolescence on hippocampal and non-hippocampal dependent tasks

To determine the effects of chronic exposure to WIN during late-adolescence on spatial learning and memory, we used two hippocampal- dependent paradigms: the aversive Morris water maze task, and the non-aversive object location task.

#### The Morris water maze

Rats were injected with vehicle or WIN for 2 weeks and after 24 h (Vehicle 24 h, n = 8; WIN 24 h, n = 7) or 10 days (Vehicle 10 d, n = 8; WIN 10 d, n = 7) taken to the water maze. The data were analyzed using a 3-way mixed ANOVA with treatment (Vehicle/WIN) and time of testing (24 h, 10 d) as between-subject factors, and the trials of training (Acq, STM, LTM, R) as a within-subject factor (**Figure 1a**).

On day one, analysis of the acquisition data [Acq_1–7_] revealed significant main effects for treatment (F_(1,26)_ = 4.03, p = 0.05), for time of testing (F_(1,26)_ = 5.31, p<0.05), and for trials (F_(1,26)_ = 112.29, p<0.001). Significant interactions were found for [trials×treatment] (F_(1,26)_ = 14.21, p = 0.01), for [trials×time of testing] (F_(1,26)_ = 12.69, p = 0.01), and for [trials×treatment×time of testing] (F_(1,26)_ = 14.43, p = 0.01). *Post-hoc* analysis revealed a significant difference in latency to find the hidden platform between the vehicle treated rats and the WIN treated rats after 24 h (p<0.05), indicating that 24 h after the last chronic injection the WIN treated rats took longer to find the hidden platform than the vehicle treated rats (**Figure 1a**).

Thirty min after training rats were tested in the maze for their short-term memory of the platform location (STM_1–3_). A significant main effect for time of testing (F_(1,26)_ = 12.3, p<0.01) was found. There was no significant main effect for treatment (F_(1,26)_<1, NS), and no significant interaction effect (F_(1,26)_<1, NS).

On day two, 48 hours after the last chronic injection, rats were tested in the maze for their long-term memory of the platform location (LTM_1–4_). A significant main effect for time of testing (F_(1,26)_ = 4.29, p<0.05) and for trials (F_(1,26)_ = 9.8, p<0.01) was found. There was no significant main effect for treatment (F_(1,26)_<1, NS), and no significant interaction effect (F_(1,26)_<1, NS). The absence of a significant difference between the treatment groups indicates that on day two there were no treatment effects on performance.

On day three, 72 hours after the last chronic injection, the platform was moved to the opposite quadrant of the maze and the rats were trained to find this new location. A significant main effect for trials (F_(1,26)_ = 128.47, p<0.001) was found. There was no significant main effect for treatment (F_(1,26)_<1, NS), for time of testing (F_(1,26)_ = 2.37, NS), and no significant interaction effect (F_(1,26)_<1, NS).

In order to find whether the pattern of acquisition on the water maze task in the WIN treated rats 24 hours after chronic treatment is the result of an acute effect on learning, we conducted a control experiment where we treated rats with an acute dose of WIN (1.2 mg/kg) and then tested their acquisition of the water maze task after 24 h (Vehicle, n = 7; Acute WIN24h, n = 7; **Figure 1a** **, inset**). Mixed design ANOVA [treatment×trials (2×7)] of the acquisition data [Acq_1–7_] revealed a significant effect for trials (F_(1,12)_ = 66.74, p<0.001), indicating the rats' improvement over trials in finding the hidden platform. There were no significant effects for treatment (F_(1,12)_<1, NS), or a significant interaction effect between trials and treatment (F_(1,12)_ = 1.73, NS). This suggests that an acute single injection of WIN does not affect the performance in the water maze task 24 h after the injection.

#### The object location task

Next, we examined the effects of chronic exposure to WIN during late-adolescence on short term spatial memory in the non-aversive object location task. The data were analyzed using a 2-way ANOVA with treatment (Vehicle/WIN) and time of testing (24 h, 10 d, 30 d, 75 d) as independent variables, and exploration index as a dependent variable. A significant main effect was found for treatment (F_(1,55)_ = 21.35, p<0.001), suggesting that in all time points measured, the WIN treated rats spent significantly less time exploring the novel location compared with the vehicle treated rats (**Figure 1b**). There was no significant main effect for time of testing (F_(3,55)_<1, NS), or a significant interaction effect (F_(3,55)_<1, NS).

One-sample t-test performed on each of the vehicle and WIN groups revealed a significant difference from the 50% exploration index point at all times tested in the Vehicle group [24 h: (t_(5)_ = 4.26, p<0.01); 10 d: (t_(8)_ = 2.82, p<0.05); 30 d: (t_(9)_ = 3.88, p<0.01); 75 d: (t_(6)_ = 2.97, p<0.05)], but not in the WIN group. Thus, 24 hours, 10, 30 and 75 days after withdrawal, rats still demonstrate impaired short-term memory in the object location task.

There was no significant difference in the sample phase (day 1) between any of the groups in exploration index (mean±SD) 24 h (Vehicle: 45.76±2.19; WIN: 51.3±1.84), 10 d (Vehicle: 52.07±4.01; WIN: 52.58±2.51), 30 d (Vehicle: 53.39±3.34; WIN: 52.86±2.11), or 75 d (Vehicle: 50.68±2.36; WIN: 51.69±1.42) after withdrawal.

In order to find whether the effects seen on spatial memory after 24 hours of withdrawal would also be evident after a single injection of WIN, we performed a control experiment, in which we examined the effects of an *acute* administration of WIN (1.2 mg/kg) on spatial short term memory in the object location task. We tested the rats in the object location task 24 hours after a single injection. Independent-samples t-test did not reveal a significant difference between the groups (Vehicle: 65.7±2.75; WIN: 68.76±2.53; t_(10)_<1, NS), indicating that an acute single injection of WIN does not affect the performance in the object location task 24 h after the injection (**figure 1b**, right square).

In a separate experiment we aimed to test whether a lower dose of chronic WIN (0.5 mg/kg instead of 1.2 mg/kg) is enough to impair performance in the object location task. As we were interested in long-term effects, rats were tested in this task after 10 days of withdrawal (Vehicle, n = 7; WIN 10 d, n = 9). Independent-samples t-test revealed that rats treated with a lower dose of WIN also exhibited impairment in short-term memory (mean±SD) (45.34±5.11) compared with vehicle treated rats (60.67±4.64), 10 days after the last injection (t_(14)_ = 2.16, p<0.05), suggesting that chronic treatment with a lower dose of WIN may be sufficient for the observed short term memory impairment.

#### The object recognition task

Since chronic exposure to WIN during late-adolescence affected non-aversive short-term hippocampal dependent spatial memory, we asked whether these effects will appear in a similar non-hippocampal dependent task. To that end we used the object-recognition task, which examines the visual and tactile properties of the explored objects rather than their spatial location.

The data were analyzed using a 2-way ANOVA with treatment (Vehicle/WIN) and time of testing (24 h, 10 d, 30 d) as independent variables and exploration index as a dependent variable (**Figure 1c**). A significant main effect was found for treatment (F_(1,50)_ = 15.17, p<0.001), and a significant interaction effect of [treatment×time of testing] (F_(2,50)_ = 3.23, p<0.05). There was no significant main effect for time of testing (F_(2,50)_ = 1.38, NS).

Independent samples t-test revealed that the main effect of treatment stemmed from the fact that the WIN treated rats spent significantly less time exploring the novel object compared with the vehicle treated rats, after 24 hrs (t_(18)_ = 3.76, p = 0.001) and 10 d (t_(10.34)_ = 2.38, p<0.05) of withdrawal.

One-sample t-test performed on each of the vehicle and WIN groups revealed a significant difference between the Vehicle group and the 50% exploration index point at all times tested, [24 h: (t_(9)_ = 6.92; p<0.001), 10 d: (t_(7)_ = 10.09; p<0.001), 30 d: (t_(9)_ = 4.89; p = 0.001)], and in the WIN group a significant difference was found at 10 d (t_(7)_ = 2.41, p<0.05) and 30 d: (t_(9)_ = 6.55, p<0.001).

In order to find whether the effects seen in the object recognition task after 24 hours of withdrawal would also be evident after a single injection of WIN, we conducted a control experiment, in which we examined the effects of an *acute administration* of WIN (1.2 mg/kg) on short term object recognition memory. Independent-samples t-test did not reveal a significant difference between the groups (Vehicle: 63.51±4.16; WIN: 60.13±3.47; t_(11)_<1, NS), indicating that an acute single injection of WIN does not affect the performance in the object recognition task 24 h after the injection (**figure 1c**, right square).

### The effects of chronic exposure to the cannabinoid receptor agonist WIN55,212-2 during late adolescence on synaptic plasticity

#### Synaptic plasticity in the perforant path-dentate gyrus pathway

Next we sought to examine whether chronic WIN administration would impair hippocampal LTP. Rats were anesthetized and taken for electrophysiological recording in the PP-DG pathway 24 h, 10 d or 30 d after the last chronic WIN injection and compared to a vehicle group. We found no difference in EPSP amplitude following HFS between vehicle injected rats tested 24 h (n = 4), 10 days (n = 4) or 30 days (n = 4) after the last injection (F_(2,9)_ = 1.2, NS), and hence the vehicle group was grouped to one for reasons of clarity.

Mixed design ANOVA [treatment×time (4×13)] post-HFS did not indicate a significant effect on EPSP amplitude for the treatment (F_(3,26)_<1, NS), or the interaction between treatment and time (F_(3,26)_ = 1.39, NS; **Figure 2a**). There was a significant within-subject effect for the time (F_(1,26)_ = 63.48, p<0.001), due to a decrease in potentiation levels from the post tetanic potentiation (measured 1 min after HFS) throughout the experiment in all groups. Hence, chronic exposure to WIN did not have a significant effect on the induction of LTP in the PP-DG pathway at any of the time points examined. Mixed design ANOVA on EPSP amplitude pre-HFS [treatment×time (4×6)] did not reveal significant effects for the treatment (F_(3,26)_ = 1.22, NS), the time (F_(1,26)_ = 2.35, NS), or the interaction between treatment and time (F_(3,26)_ = 2.72, NS).

#### Synaptic plasticity in the ventral subiculum-nucleus accumbens pathway

Rats were anesthetized and taken for electrophysiological recording in the vSub-NAc 24 h, 10 d or 30 d after the last WIN injection and compared to a vehicle group. We found no difference between vehicle injected rats tested 24 h, 10 d or 30 d after the last injection, and hence the vehicle group was grouped to one for reasons of clarity (n = 8).

Mixed design ANOVA [treatment×time (4×13)] post-HFS indicated significant effects on EPSP amplitude for the treatment (F_(3,28)_ = 6.91, p = 0.001), for the time (F_(1,28)_ = 4.18, p = 0.05), but not for the interaction between treatment and time (F_(3,28)_<1, NS; **Figure 2b**). *Post hoc* analysis revealed significantly higher levels of potentiation in the vehicle group and the WIN 30 d group compared with the WIN 24 h group (p<0.01, different from Vehicle; p<0.05, different from WIN 30 d) and with the WIN 10 d group (p<0.001, different from Vehicle; p<0.01, different from WIN 30 d). Hence, chronic exposure to WIN impaired the induction of LTP in the vSub-NAc pathway 24 h and 10 d, but not 30 d after withdrawal. Mixed design ANOVA on EPSP amplitude pre-HFS [treatment×time (4×6)] did not reveal significant effects for the treatment (F_(3,28)_<1, NS), or the time (F_(1,28)_<1, NS), or the interaction between treatment and time (F_(3,28)_ = 1.85, NS).

In order to find whether the impairment of LTP seen after 24 h and 10 d would appear also following a single injection of WIN, rats were treated with an acute dose of WIN (1.2 mg/kg) and then taken for electrophysiological recording in the vSub-NAc after 24 h (Vehicle, n = 5; Acute WIN24h, n = 5; **Figure 2b** **, inset** on the right side). Mixed design ANOVA [treatment×time (2×13)] indicated significant effects on EPSP amplitude for the treatment (F_(1,8)_ = 17.51, p<0.01), for the time (F_(1,8)_ = 8.9, p<0.05), but not for the interaction between treatment and time (F_(1,8)_<1, NS). This suggests that an acute single injection of WIN affects LTP in the NAc 24 h after the injection. Mixed design ANOVA on EPSP amplitude pre-HFS [treatment×time (2×6)] did not reveal significant effects for the treatment (F_(1,8)_<1, NS), or the time (F_(1,8)_<1, NS), or the interaction between treatment and time (F_(1,8)_<1, NS).

### The effects of chronic exposure to the cannabinoid receptor agonist WIN55,212-2 during late adolescence on the rats' weight, sucrose consumption and performance in an open field test

To exclude motor deficits or other non-specific alterations that might have caused the effects on learning and plasticity, rats were chronically administered with WIN and tested for locomotion and anxiety levels in the open field and their weights and sucrose consumption were monitored.

#### Open field

The data were analyzed using a 2-way ANOVA with treatment (Vehicle/WIN) and time of testing (24 h, 10 d, 30 d) as independent variables, and time in the center/number of rearings/distance covered as dependent variables.

Analysis of the time the rats spent in the center of the open field revealed a significant main effect for time of testing (F_(2,47)_ = 5.39, p<0.01), a significant effect for treatment (F_(1,47)_ = 5.6, p<0.05), and a significant interaction effect (F_(2,47)_ = 3.85, p<0.05). Post-hoc comparisons revealed that the significant main effect of time of testing stemmed from a significant difference between the 24 h groups and the 10 d groups (p<0.01), and between the 24 h group and the 30 d group (p<0.01).

Independent-samples t-test revealed a significant difference between the treatment groups 24 h after withdrawal (t_(12.83)_<1, p = 0.01), suggesting that the WIN group (n = 10) spent significantly more time in the center of the open field than the vehicle group (n = 10), perhaps indicating their lower level of stress compared to the vehicle group (**Figure 3a**). There were no significant differences between the treatment groups after 10 days of withdrawal (t_(17)_<1, NS), or after 30 days of withdrawal (t_(8.8)_<1, NS).

Analysis of the number of rearings the rats performed in the open field revealed a significant main effect for time of testing (F_(2,47)_ = 20.78, p<0.001; **Figure 3b**). Post-hoc comparisons revealed that this effect stems from significant differences between the 24 h groups (mean±SD) (WIN: 35.8±2.94; Vehicle: 32.4±2.94) and the 10 d groups (WIN: 16.56±3.1; Vehicle: 18.6±2.94; p<0.001), and between the 24 h group and the 30 d group (WIN: 12.57±3.5; Vehicle: 20.43±3.52; p<0.001).

Analysis of the distance the rats covered in the open field revealed a marginally significant main effect for treatment (F_(1,46)_ = 3.96, p = 0.053). However, independent-samples t-test did not reveal a significant difference between the treatment groups at any of the time points tested (24 h: t_(18)_<1, NS; 10 d: t_(16)_<1, NS; 30 d: t_(12)_<1, NS), suggesting no effect on gross motoric behavior (**Figure 3c**). This could indicate a possible tolerance effect to the chronic administration of WIN.

#### Sucrose consumption test

To examine the effects of chronic exposure to WIN during late-adolescence on hedonia, we used the sucrose consumption test. We measured sucrose intake before injection (baseline), 24 h, 10 d, and 30 d after the last injection (**Figure 4a**).

Two-way ANOVA with treatment (Vehicle/WIN) and time of testing (baseline, 24 h, 10 d, 30 d) as independent variables, and sucrose consumption as the dependent variable did not reveal significant effects for the treatment (F_(1,13)_<1, NS), or the interaction between treatment and time of testing (F_(1,13)_<1, NS). There was a significant main effect for time of testing (F_(1,13)_ = 9.51, p<0.01).

#### Weight

We also measured the rats' weight up to 30 days after the last injection. Rats were weighed before injection (baseline), 24 hrs, 10 days, or 30 d after the last injection (**Figure 4b**). Mixed design ANOVA on weight gain [treatment×time point (2×4)] did not reveal significant effects for the treatment (F_(1,11)_ = 3.11, NS), or the interaction between treatment and time point (F_(1,11)_<1, NS), but there was a significant within-subject effect for the time point (F_(1,11)_ = 660.98, p<0.001), signifying the rats' weight gain over time.

## Discussion

Our findings suggest that the effects of chronic cannabinoid exposure in the late-adolescent period in rats on learning and memory are task- and brain region-specific (see **Table 1**). The most robust effect of chronic WIN administration was the impairment of short-term memory in the spatial version of the object recognition task that persisted even after 75 days of withdrawal. However, we found a gradual recovery of behavioral and electrophysiological impairments in acquisition and short-term memory in the water maze, short-term object recognition memory, and LTP in the vSub-NAc pathway. No significant long-term effects of chronic WIN were observed on locomotion, sucrose consumption or weight gain. Hence, most of the deficits observed were temporary corroborating with previous studies showing that long-term cannabinoid administration produces CB1 receptor desensitization and down-regulation in the hippocampus that recovers to control level at 14 days after cessation of treatment [39], [40].

**Table 1:** Summary of results.

	Time Point		
Test	24 hours	10 days	30 days
**Water maze**	Impairment in acquisition	No effect	-
**Location recognition**	Impairment in short-term memory	Impairment in short-term memory	Impairment in short-term memory (also at 75 days)
**Object recognition**	Impairment in short-term memory	Attenuation of short-term memory	No effect
**Synaptic plasticity – PP-DG**	No effect	No effect	No effect
**Synaptic plasticity – vSub-NAc**	Impairment of LTP	Impairment of LTP	No effect
**Open field**	Increased time in the center	No effect	No effect
**Sucrose consumption**	No effect	No effect	No effect
**Weight**	No effect	No effect	No effect

The table summarizes the effects of chronic i.p. treatment with the CB1/CB2 receptor agonist WIN55,212-2 (1.2 mg/kg) for two weeks during the late adolescence period (post-natal days 45–60) on behavioral and electrophysiological measures of cognitive performance tested 24 hrs, 10 and 30 days after the last drug injection.

### The effects of chronic exposure to WIN55,212-2 during late adolescence on performance in behavioral tasks

The acquisition in the spatial task in the Morris water maze was impaired after 24 hrs of withdrawal corroborating with previous studies using THC and HU-210 [41], [42]. The impairment was no longer evident after 10 days of withdrawal, suggesting that the 24 hrs effect could be due to drug residue of cannabinoids in the CNS or to withdrawal effects from the drug. This is consistent with Wise et al. [43], showing that the induction of withdrawal in THC-dependent rats impaired performance in the water maze. Twenty-four hours after withdrawal, both treatment groups showed poor short-term spatial memory, perhaps due to the less efficient training in the massed protocol compared with the spaced protocol used in other studies [33]. Acute treatment with WIN did not affect the acquisition of the water maze task, excluding the possibility that an acute single injection of WIN affects spatial learning 24 h after the injection.

In the spatial version of the object recognition task, WIN-treated rats showed impairment in short-term memory that was evident up to 75 days after the last injection. This is a robust effect suggesting that this type of spatial memory is more sensitive to the effects of chronic WIN exposure during late adolescence than the water maze task. The first explanation for the differential effects of WIN on performance involves the different level of stress in each task. The water maze is a highly aversive learning task, especially when using the massed training protocol [33] whereas the object location task is considered non-aversive. The cannabinoid system and the stress system are highly interconnected [44]–[49] and it may be that the effects of WIN on performance in a stressful learning paradigm are different than in a neutral task. Several studies suggested that the cannabinoid system is not involved in the extinction of non-aversive memories [50]–[52]. Harloe et al. [50] examined extinction learning under aversive and appetitive conditions, and reported impairment of extinction learning by a cannabinoid antagonist only under aversive conditions, suggesting that the endocannabinoid system might become activated specifically in highly aversive situations. Similarly, we have recently shown that WIN microinjected into the basolateral amygdala can prevent the effects of stress exposure (elevated platform stress) on performance in an aversive learning task (i.e., conditioned avoidance and extinction of inhibitory avoidance) [53], but WIN could not prevent the effects of the same stressor on the performance in the non-aversive object location task [54]. Hence, a possible interaction between WIN and the stressfulness of the task may have a different outcome on performance. The second explanation relates to the brain areas involved in these tasks. The water maze task heavily relies on the dorsal CA1 area [55]–[57], and the object location task, although heavily relying on the hippocampus, also involves other brain areas (i.e., prefrontal cortex, perirhinal cortex; [58]–[61], [38]) that are also affected by the chronic systemic administration of WIN. As in the water maze task, chronic treatment with WIN was necessary in order to cause impairment in performance, since no impairment was evident 24 hours after a single WIN injection.

In the non-spatial object recognition task, we found a gradual recovery over time of short-term memory impairment, perhaps due to drug residue. At 10 days after withdrawal, the WIN treated rats demonstrated attenuated performance compared to the vehicle treated rats, however their exploration index was significantly higher than chance levels, suggesting that they acquired the task. After 30 days of withdrawal, when the drug components would reasonably be expected to have disappeared from the CNS [62], [63], no effect on short-term memory could be discerned. The object recognition task is to a great extent dependent on the prefrontal cortex and the perirhinal cortex [37], [38], which may suggest a greater sensitivity of the hippocampus than the cortex to the effects of chronic WIN treatment during late-adolescence. An acute single injection of WIN did not affect performance in the object recognition task 24 h after the injection suggesting that chronic treatment with WIN is required for memory impairment to occur.

### The effects of chronic exposure to WIN55,212-2 during late adolescence on synaptic plasticity

Chronic WIN administration during late adolescence had no significant effect on plasticity in the DG hippocampal area although there are very high levels of CB1 receptors in all subfields of the hippocampus, including the DG [64]–[66]. A possible explanation for the lack of effect is low sensitivity or adaptation of DG neurons to high chronic levels of WIN. Yet, a recent study found that acute WIN affects miniature inhibitory postsynaptic currents in the DG without altering event amplitude, area, rise time, or decay [67]. This study showed that WIN potentiated action potential-independent release of GABA in the DG which was not mediated through a CB1or CB2 receptor mechanism [67]. This may suggest that although WIN has no effect on HFS-induced plasticity in the DG, it affects the spontaneous release of GABA.

We have recently found that *acute* administration of WIN (0.5 mg/kg) significantly impaired LTP in the schaffer collateral-CA1 projection [12]. Here we found that chronic administration of either *vehicle* or WIN impaired LTP in the schaffer collateral-CA1 pathway (data not shown). Hence, we could not differentiate, using our paradigm, between the effects of chronic WIN exposure and the stress-induced effects of chronic i.p. injections on LTP. Daily injections can constitute chronic stress for the rats, and the CA1 area is highly sensitive to stress [68], [69]. Hill et al. [70] found that rats that were chronically exposed to high levels of the CB1 receptor agonist HU-210 demonstrate impaired LTP in the CA1 region when examined 18 h following the final drug administration. There are several differences between our experiment and Hill's; drug administration began when the rats were 300 g, the same point at which we end our drug administration; electrophysiological recording and stimulating electrodes were in different coordinates than in our chronic experiment; rats injected with HU-210 were taken for electrophysiology after completing extensive behavioral training.

In the vSub-NAc pathway chronic WIN impaired LTP induced after 24 hrs or 10 days, but not after 30 days, of withdrawal. LTP in this pathway is NMDA-dependent [71], [72]. Also, it has been suggested that the NAc, by integrating vSub input of contextual information, mediates goal-directed behavior and that the hippocampal input in the NAc is well placed to influence reward and incentive systems [73], [74]. The NAc represents a critical site for mediating the rewarding and/or addictive properties of several classes of abused drugs, including ethanol, opioids, psychomotor stimulants, and marijuana [75]–[77].

Acute treatment with WIN impaired LTP after 24 hours in this pathway. This could suggest that the NAc is particularly sensitive to the effects of WIN due to its involvement in the neural processing of rewarding stimuli. The role of the NAc in behavior reinforced by both natural reward [78] and drugs of abuse is supported by vast experimental evidence [79], [80]. Moreover, in vivo experiments indicate that cannabinoids reduce excitability of NAc neurons [81]. This acute effect is consistent with Mato et al. [82] who found impairment of LTD in the NAc and CA1, 24 hours following a single *in vivo* injection of THC. This effect was reversible within 3 days, suggesting that the modification in the functional properties of cannabinoid receptors was transient. Together with our previous findings on the impairing effects of acute WIN on LTP in the CA1 [12] the data suggest that alterations in synaptic plasticity as a result of cannabinoid treatment can occur within 24 hours of acute exposure.

### The effects of chronic exposure to WIN55,212-2 during late adolescence on non memory related measures

Chronic exposure to WIN had no effect on weight gain, sucrose consumption or gross locomotion, but it significantly affected the rats' level of anxiety, as measured in the open field, after 24 hrs of withdrawal. These findings suggest that the long-term effects of chronic WIN on learning and plasticity are probably not due to changes in sensory-motor parameters or other non-specific effects.

Since acute administration of cannabinoids results in depression of motoric activity in the open field, and chronic administration results in tolerance to these effects [83], the lack of effect here on locomotion could be the result of tolerance to WIN, developed over the two weeks of daily injections.

Increased time spent in the central part of the open field arena is an indication of a reduction in anxiety-like behavior [84]. Activation of the cannabinoid system has anxiolytic properties [45], [85] that could explain the effect in the open field 24 hrs after withdrawal when drug residue may still be present in the CNS.

Anhedonia, or the decreased ability to experience pleasure, can be examined in rats by reduction in sucrose intake. Responding to natural and artificial rewards is mediated by the NAc and its dopaminergic inputs [86]. Although we found that chronic WIN interferes with LTP in the NAc, no effect was observed in sucrose consumption. This may suggest a greater sensitivity of the LTP-mediated neural circuit in the NAc to the effects of chronic WIN than the neural circuit mediating sucrose intake.

### Long-term effects of cannabinoids

Prolonged exposure to cannabinoid agonists in laboratory animals is associated with the development of tolerance to most of their pharmacological effects [87]. There is a brief ‘drug residue’ effect of 12–24 hrs after acute exposure to cannabinoids that may persist longer in chronic users [63]. The average terminal elimination half-life of the THC metabolite THCCOOH in plasma of chronic cannabis users is as long as 4.3 days and may be as long as 12.6 days [88]. WIN has a shorter half-life than THC [89]–[91] and undergoes significant metabolism similar to that of other cannabinoids [92]. Prolonged treatment with THC or with WIN resulted in cannabinoid receptor desensitization and down-regulation throughout the brain, as well as tolerance to cannabinoid-mediated effects, and attenuation of CB1 receptor-mediated G-protein activation that persisted for several days after cessation of treatment [39]–[40]. In adolescent rats, this desensitization of CB1 receptors following prolonged treatment with THC is slower than in adults, perhaps contributing to the differentiation in long-lasting cognitive effects between adolescents and adults [93].

### Summary

Our results point to a gradual recovery over time rather than persistent long-lasting impairments following chronic WIN administration. Yet, WIN had a long-term impairing effect on performance in a non-aversive hippocampal-dependent short-term memory task, corroborating animal and human studies on short-term memory [94]–[98].

Studying the lasting effects of cannabinoids on cognitive function may advance our understanding of the potential harmful consequences of cannabinoids. Dissociating the short-term from the long-lasting effects of cannabinoids may indicate whether long-term exposure to cannabinoids is associated with long-lasting deficits in higher brain function that persist after drug use stops. This will help in determining whether the clinical benefits of using cannabinoids outweigh the risks, and to better cope with the deficits induced by cannabinoids.
